# Calcium-Enhanced Medium-Based Delivery of Splice Modulating Antisense Oligonucleotides in 2D and 3D hiPSC-Derived Neuronal Models

**DOI:** 10.3390/biomedicines12091933

**Published:** 2024-08-23

**Authors:** Ronald A. M. Buijsen, Linda M. van der Graaf, Elsa C. Kuijper, Barry A. Pepers, Elena Daoutsali, Lotte Weel, Vered Raz, David A. Parfitt, Willeke M. C. van Roon-Mom

**Affiliations:** Department of Human Genetics, Leiden University Medical Center, 2333 ZA Leiden, The Netherlands

**Keywords:** ASO delivery, organoids, CEM

## Abstract

Antisense technology demonstrates significant potential for addressing inherited brain diseases, with over a dozen products already available and numerous others in the development pipeline. The versatility of differentiating induced pluripotent stem cells (iPSCs) into nearly all neural cell types proves invaluable for comprehending the mechanisms behind neurological diseases, replicating cellular phenotypes, and advancing the testing and development of new therapies, including antisense oligonucleotide therapeutics. While delivering antisense oligonucleotides (ASOs) to human iPSC-based neuronal models has posed challenges, this study explores various delivery methods, including lipid-based transfection, gymnotic uptake, Ca^(2+)^-enhanced medium (CEM)-based delivery, and electroporation, in 2D and 3D hiPSC-derived neuronal models. This study reveals that CEM-based delivery exhibits efficiency and low toxicity in both 2D neuronal cultures and 3D brain organoids. Furthermore, the findings indicate that CEM is slightly more effective in neurons than in astrocytes, suggesting promising avenues for further exploration and optimization of preclinical ASO strategies in the treatment of neurological disorders.

## 1. Introduction

Antisense technology has shown promise to revolutionize the treatment of inherited diseases. To date, there are over a dozen antisense technology products on the market, and many others are being developed by academic institutes and pharmaceutical companies. Synthetic antisense oligonucleotides are single- or double-stranded strings of modified nucleic acids that can be used as therapeutic agents in different ways, but all these modalities bind to their specific target via Watson–Crick–Franklin base pairing [1]. The use of antisense technology as a promising therapeutic platform for neurological diseases was shown by the approval of nusinersen for the treatment of spinal muscular atrophy (SMA) patients by both the Food and Drug Administration (FDA) and the European Medicines Agency (EMA) [2]. Nusinersen is an 18-mer 2′-MOE phosphorothioate ASO that acts as a splice-altering ON designed to allow for a more complete translation of SMN protein from the paralogous gene *SMN2*. Treatment of SMA patients is successful by slowing disease progression and improving disease symptoms. Various antisense therapeutics for several other neurological conditions, including amyotrophic lateral sclerosis (ALS), Huntington’s disease (HD), different forms of spinocerebellar ataxia (SCA), and Alzheimer’s disease (AD), are currently being tested in clinical trials [3].

For preclinical testing of ASO intervention strategies, many different preclinical cell and animal models are used. Current preclinical models are often not patient-derived and do not express the mutation in the gene of interest. Moreover, since ASOs target human RNA transcripts, it is important to have a highly expressed human transcript present to perform a first preclinical screening. The discovery of reprogramming somatic cells into human induced pluripotent stem cells (hiPSCs) has revolutionized the modeling of human diseases, including neurological disorders [4,5]. Human iPSCs provide the ability to capture the heterogeneity that develops from gender, ethnicity, and biological variability specific to the patients from which they have been derived [6]. The ability to differentiate iPSCs into almost all neural cell types provides a valuable tool for understanding neurological disease mechanisms, replicating cellular phenotypes, and developing and testing new therapies, including ASO therapeutics [7,8]. Furthermore, differentiating iPSCs into neural cells is useful since some disease-relevant genes may be specifically expressed in particular (neural) cell types. A disadvantage is that these monolayer cell models lack the cytoarchitectural structures needed for cellular interactions. Brain organoids generated from hiPSCs have the ability to self-organize to specific brain structures and are an ideal model for studying both cellular interactions and the pathophysiology of the human brain [9]. Organoids provide an innovative platform for drug discovery in neurological disease and have successfully been used to test small molecules and repurposed drugs. Currently, human brain organoids are increasingly used in modeling neurological disorders and therapeutic discovery studies [10,11].

Standard, lipid-based ASO transfection protocols are useful for studying dose–response effects in easy-to-culture cells but seem to be toxic in hiPSC-derived neuronal cultures. It has been reported that Ca^(2+)^ enhanced medium (CEM) potentiates the activity of multiple types of naked oligonucleotides in various cultured cell lines and with limited cytotoxicity [12]. Moreover, it has been shown that the in vitro potency of ASO treatment in cells using CEM has a higher positive correlation with in vivo activity compared to conventional delivery methods. Another strategy is the delivery of ASOs to cells in the absence of any carriers or conjugation, so-called gymnotic delivery. To date, only one study has used gymnotic ASO delivery in brain organoids, and this procedure can benefit from optimization [13]. Here, cortical organoids derived from iPSCs from frontotemporal dementia patients were successfully treated with a high concentration of ASO. These results are limited to this particular experiment since extremely high concentrations of the ASO were used, which are not comparable to the physiological concentrations. However, this is a promising start for the use of brain organoids to study novel ASO strategies.

In this study, a screening method to analyze lipid-based transfection, gymnotic uptake, CEM-based delivery, and electroporation methods for ASOs was tested in 2D and 3D hiPSC-derived neuronal models. Gymnotic uptake was not efficient in the concentrations used, but CEM resulted in high transfection efficiency and low toxicity in 2D neuronal cultures. We showed in these 2D neuronal cultures that CEM is slightly more efficient in neurons compared to astrocytes. In two-week-old 3D brain organoids, increasing ASO concentrations resulted in an increased ASO efficiency. Furthermore, we found that CEM has a longer effect than gymnotic uptake and electroporation. Altogether, CEM is an efficient and low-toxic ASO delivery method in iPSC-derived neuronal 2D cultures and 3D brain organoids.

## 2. Materials and Methods

### 2.1. Neural Progenitor Cell Cultures

Control NPCs from a previous study were used [14]. Neural progenitor cells (NPCs) were cultured following standard protocols (STEMCELL technologies). A total of 2 × 10^6^ NPCs were defrosted into 6-well plates coated with Matrigel, expanded, and passed into 6-well plates coated with Poly-D-lysine (PDL)/laminin for further use in neuronal differentiation and maturation.

### 2.2. Neural Differentiation and Maturation

A total of 750,000 NPCs were seeded into a well of a 6-well plate coated with PDL/laminin and neuronally differentiated according to standard protocols using a STEMdiff Forebrain Neuron Differentiation Medium kit (STEMCELL Technologies, Vancouver, BC, Canada). The medium was changed daily with STEMdiff Forebrain Neuron Differentiation Medium until day 7.

After 7 days, the maturation process was started using the BrainPhys hPSC Neuron kit from STEMCELL Technologies. Cells were seeded on a 96-well or 12-well plate coated with PDL/laminin and incubated with respectively 17,000 or 100,000 cells per well in 50 or 500 µL medium. The next day, complete BrainPhys Neuronal medium was added to each well, and half-medium changes were performed every 2–3 days, which was continued for 2 weeks. Transfections were performed on day 11 of maturation and harvested on day 14 of maturation.

### 2.3. Organoid Preparation

Organoids were generated using the STEMdiff™ Cerebral Organoid Kit (STEMCELL Technologies) according to the protocol. ASO uptake experiments were performed on day 14 or 35 of the standard protocol.

### 2.4. Calcium Chloride-Enhanced Medium (CEM), Gymnotic Uptake, and Lipofectamine-Mediated ASO Uptake in 2D Neuronal Cell Culture

Complete BrainPhys Neuronal medium was supplemented with 9 mM calcium chloride (CaCl_2_) and 2% FBS (for CEM-mediated uptake) or extra BrainPhys medium (gymnotic ASO uptake).

For Lipofectamine transfection, cells were incubated in DMEM/F12. ASO suspension was made with 500 nM ASO and 2.5 µL Lipofectamine 2000 incubated in 50 µL DMEM/F12 for 5 min. Then, the ASO suspension was added to the Lipofectamine mixture and incubated for another 20 min. One hundred µL Lipofectamine/ASO mix was added to the cells and incubated at 37 °C for 4 h, after which the medium was changed to complete BrainPhys Neuronal medium and incubated for a total of 72 h.

Cells were harvested by washing with DMEM/F12 and incubated for 5 min with Accutase (Gibco) at 37 °C. DMEM/F12 was added, and the cell suspension was pipetted into a 15 mL tube. After spinning down and washing with PBS, the supernatant was discarded, and the cell pellet was frozen at −20 °C until RNA isolation. The ASOs are shown in Table 1.

### 2.5. Antisense Oligonucleotides

All ASOs were synthesized using standard solid-phase phosphoramidite chemistry according to previously published protocols (Capaldi and Scozzari, 2007) [15] and purified using AEX-HLPC.

### 2.6. NEPA 3D Electroporation

Organoids were washed with PBS twice and collected in 100 µL Opti-MEM™ plus ASO. Electroporation parameters were as shown in Table 2; the cuvette was placed into the CU500 Cuvette Chamber, and the electroporation program was executed. To avoid cell damage, organoid medium was added into the cuvette right after the electroporation, and the organoids with the medium were placed in 24-well cell culture plates.

### 2.7. Calcium Chloride-Enhanced Media (CEM)-Mediated ASO Uptake in 3D Neuronal Cell Culture

For 3D organoid CEM-mediated ASO uptake, 2 mL of maturation medium, enhanced with 9 mM CaCl_2_ and 2% FBS per well, was put in a 12-well plate. Four to six 29-day-old organoids were added per well. ASO HTT-AON12.1 or APP-AT040 ASO was added per well in an end concentration of 100,000 nM. Organoids were incubated for 72 h, and medium changing every 3–4 days continued. At 1 week, 2 weeks, and 4 weeks after ASO treatment, 2 organoids per condition were washed in PBS and frozen in an Eppendorf tube until RNA isolation. To assess transfection efficiency, pictures of the H40-fluorescent signal in the organoids were made with an Invitrogen™ EVOS™ FL Digital Inverted Fluorescence Microscope.

### 2.8. RNA Isolation, cDNA Synthesis, and RT-PCR

RNA isolation was performed using the ReliaPrep^TM^ Miniprep System (Promega, Madison, WI, USA). A total of 500 ng RNA was used per reaction for cDNA synthesis, using the transcriptor first strand cDNA synthesis kit (Roche, Basel, Switzerland). The skip of the exon was checked by RT-PCR. For the HTT-AON12.1, the cycle parameters were 4 min at 95 °C and 35 cycles of 30 s at 95 °C, 30 s at 59 °C and 70 s at 72 °C, ended by 7 min at 72 °C. For the APP-AT040 ASO, the cycle parameters were 4 min at 95 °C and 40 cycles of 10 s at 95 °C, 30 s at 57 °C and 20 s at 72 °C, ended by 7 min at 72 °C. The PCR products were run on a 2% agarose gel. The primers are listed in Table 3.

### 2.9. Immunocytochemistry

Immunocytochemistry was performed using standard protocols. In short, cells were fixed with 4% Paraformaldehyde (PFA) and washed in PBS with 0.1% triton three times for 5 min. Cells were incubated with primary antibody overnight at 4 °C in immunobuffer (1× PBS with 0.1% triton, 1% normal goat serum, and 0.04% merthiolate). After incubation, cells were washed for 5 min with PBS three times and incubated with the secondary antibody in immunobuffer for 3 h at room temperature. Cells were washed again three times in PBS and mounted. To check whether the stainings were successful, the EVOS microscope was used. For details on the antibodies and dilutions, see Table 4.

### 2.10. Organoid Embedding

Organoids for immunohistochemistry were washed in PBS and fixed in 4% PFA for 15 min. After a PBS washing step, organoids were stored at 4 °C in an Eppendorf tube with 30% sucrose overnight or up to 7 days until they sunk to the bottom of the tube. Next, organoids were transferred to a sucrose solution with trypan blue added in a concentration of 1:50 to be able to see the embedded organoids during cutting and incubated at room temperature for 10 min. OCT Mounting Medium was put in a T12 embedding mold (Polysciences Inc., Warrington, PA, USA), and 2–3 organoids were placed together into 1 embedding mold. The mold with organoids was placed in a 100% ethanol/dry ice freezing bath on top of dry ice for at least 1 min to shock-freeze the Tissue Tek. Blocks were stored at −80 °C until cutting. 

Organoids were cut with a cryotome machine at 16 µm, placed on Superfrost Plus slides (Fisher Scientific, Waltham, MA, USA), and stored at −20 °C for immunohistochemistry.

### 2.11. Immunohistochemistry on Organoid Sections

Slides with organoid sections were defrosted for 30 min at room temperature. Sections were washed in PBS and incubated with immunobuffer for 1 h at room temperature. Sections were then incubated with the primary antibody in immunobuffer overnight at 4 °C. After incubation, slides were washed three times 5 min in PBS, and the secondary antibody was incubated for 2 h at room temperature. Slides were again washed three times 5 min in PBS and mounted with a coverslip and mounting medium. Images were obtained on a Leica TCS SP8 microscope. For details on the antibodies and dilutions, see Table 4.

### 2.12. Transfection Efficiency of Antisense Oligonucleotide

To investigate the efficiency of antisense oligonucleotide (ASO) delivery, 96-well plates with iPSC-derived neuronal cultures were stained for the neuronal marker microtubule-associated protein 2 (MAP2), the astrocyte marker glial fibrillary acidic protein (GFAP) (Invitrogen, Carlsbad, CA, USA) and DAPI (Sigma Aldrich, Burlington, MA, USA) as performed previously (Table 4 and [14]). Plates were scanned with the Cellomics HTC platform (Thermofisher, Waltham, MA, USA) on a 20× magnification and analyzed with the HCS Studio Cell Analysis software (https://www.thermofisher.com/nl/en/home/life-science/cell-analysis/cellular-imaging/hcs-hca.html?gclid=EAIaIQobChMIrc2p1ZiGiAMVupWDBx10BCAzEAAYASAAEgJ5yPD_BwE&ef_id=EAIaIQobChMIrc2p1ZiGiAMVupWDBx10BCAzEAAYASAAEgJ5yPD_BwE:G:s&s_kwcid=AL!3652!3!568311315550!p!!g!!automated%20cell%20analyzer!6675222851!82804340561&cid=bid_pca_cx7_r01_co_cp1359_pjt0000_bid00000_0se_gaw_nt_lgn_ins&gad_source=1, accessed on 20 August 2024, Thermo Scientific, Waltham, MA, USA). The maximum number of fields per well was set at 41. For the settings, see Table 5. 

First, the nuclei (Ch1) were segmented, and invalid objects were excluded; subsequently, the ASO (Ch2) signal was segmented. To investigate how many nuclei contain ASO, the colocalization toolbox was used, where the DAPI channel was selected as the region of interest A (ROIA). The FAM label in channel 2 was selected as target 1. The feature of ROIA_Target1 reported percentages of cells positive for ASO in the nucleus. 

For the analysis of cell-type-specific ASO transfection efficiency, the colocalization toolbox was applied. Neurons were segmented based on MAP2 staining. After identification and validation, the neurons were chosen as region of interest B (ROIB) and ASO (ch 2) as target 1. The overlap of ROIB with ROIA (nuclei) was selected in order to run the analysis only on the neuronal nuclei. The ASO (Ch2) was set as target 1 of ROIB, and the output feature ROIB_Target1 therefore gave the percentages of neurons that contained ASO. The same process was repeated for astrocytes, which were stained with GFAP.

## 3. Results

### 3.1. CEM Improves Survival in hiPSC-Derived Neuronal Cultures

**Figure 1:**
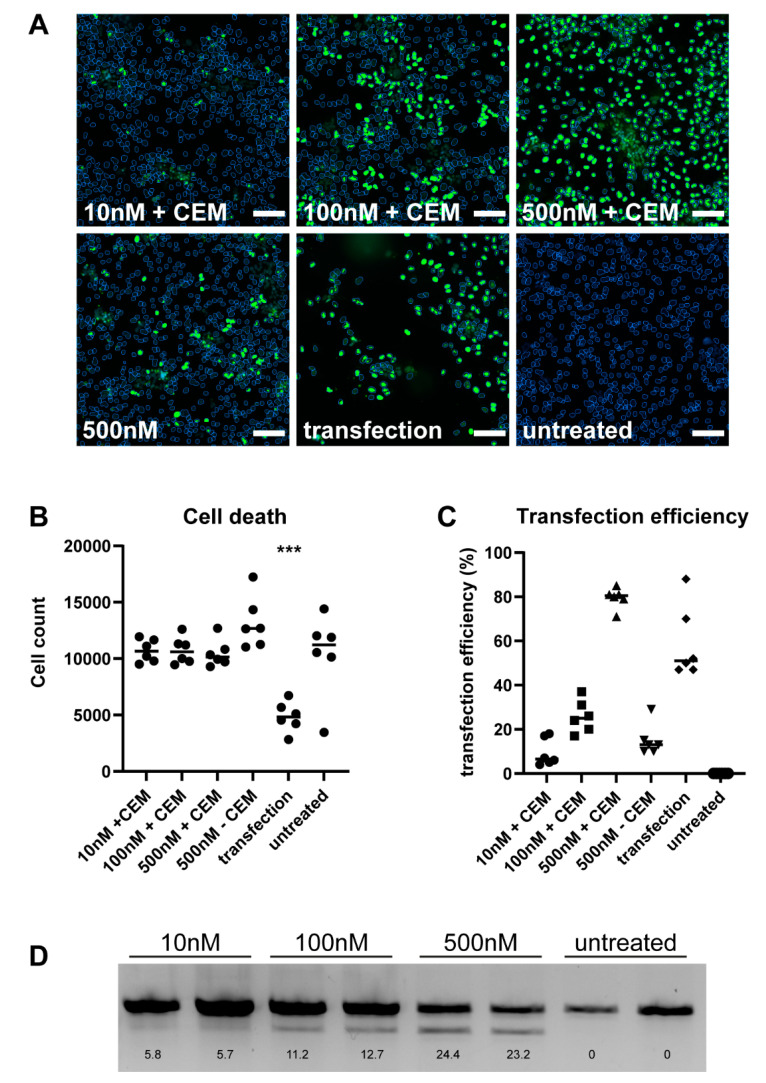
CEM improves survival in 2D hiPSC-derived neuronal cultures. (**A**) Representative images of iPSC-neurons treated with increasing concentrations of FAM-ASO (green) with CEM, compared to no CEM or Lipofectamine transfection as controls. Untreated hiPSC neurons are shown as negative control. The scale bar represents 25 µm. (**B**) Quantification of number of nuclei per condition and (**C**) transfection efficiency represented by the percentage of FAM-positive nuclei using the Cellomics high-content imaging platform. *n* = 6. (**D**) Dose–response analysis of hiPSC neurons showing increased splicing with increasing concentration of AON12.1 with the exon-skip percentages indicated at the bottom of each lane. *** *p* < 0.001.

Lipofectamine transfections in iPSC-derived neuronal cultures may cause cell death, so we investigated the efficiency and toxicity of ASO delivery using calcium-enhanced medium (CEM) (Figure 1A,B). When we treated iPSC-derived neuronal cultures with increasing concentrations of a FAM-labeled ASO using gymnotic uptake, CEM, and lipofectamine, there was a significant reduction in the number of nuclei only after lipofectamine transfection (Figure 1A,B). Quantification of the transfection efficiency represented by the number of FAM-positive nuclei showed an increasing number of FAM-positive nuclei with increasing concentrations of ASO in the CEM condition. Furthermore, CEM is more efficient than lipofectamine transfection (*p* = 0.0032, Tukey’s multiple comparisons test) (Figure 1C). Previously, we have shown in a proof-of-concept using antisense oligonucleotides to induce skipping of exon 12 in huntingtin (*HTT*) pre-mRNA, thereby preventing the formation of a 586 amino acid N-terminal huntingtin fragment implicated in HD toxicity [16]. To functionally test CEM delivery, we delivered an increasing dose of HTT-AON12.1 in iPSC-derived neuronal cultures and observed a clear dose effect in exon-skip efficiency. The lower band in the PCR reaction is an in-frame partial exon 12 skip in human Htt pre-mRNA ([16] and Figure 1D).

### 3.2. CEM Delivery Is More Efficient in Neurons Compared to Astrocytes

**Figure 2:**
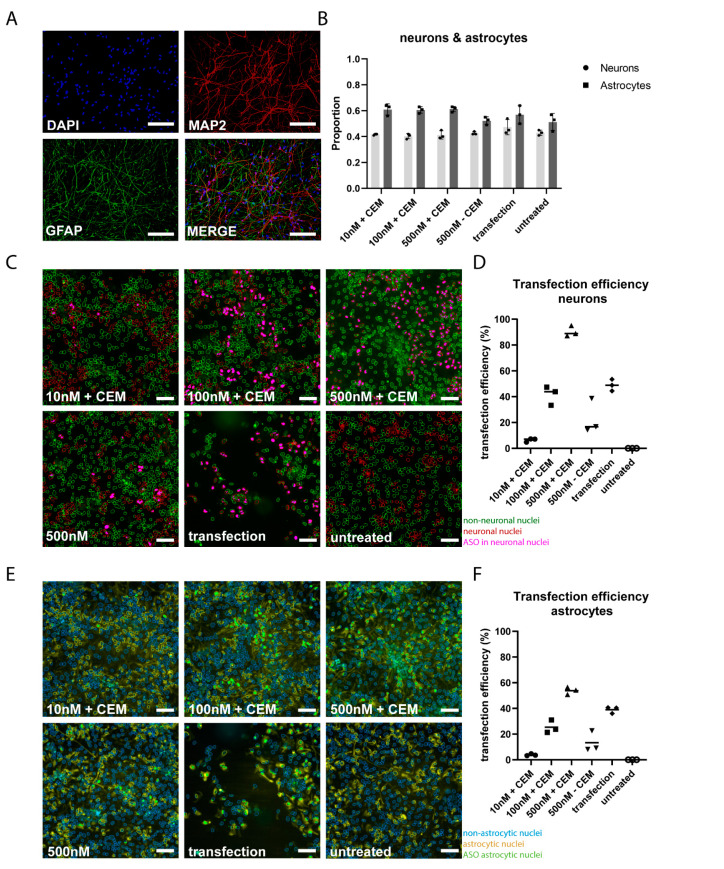
AON delivery using CEM is more efficient in neurons compared to astrocytes in 2D hiPSC-derived neuronal cultures. (**A**) Representative images of hiPSC neurons stained with the neuronal marker MAP2 and the astrocyte marker GFAP. The scale bar represents 25 µm. (**B**) Quantification of proportion neurons and astrocytes using Cellomics high-content imaging platform. *n* = 3. (**C**) Representative images of iPSC-neurons treated with increasing concentrations of FAM-AON using CEM, compared to no CEM or Lipofectamine as controls. Untreated hiPSC neurons are shown as negative control. The scale bar represents 25 µm. (**D**) Quantification of transfection efficiency in neurons using Cellomics high-content imaging platform. *n* = 6. (**E**) Representative images of hiPSC neurons treated using increasing concentrations of FAM-AON with CEM compared to no CEM or Lipofectamine as controls. Untreated hiPSC neurons are shown as negative control. The scale bar represents 25 µm. (**F**) Quantification of transfection efficiency in astrocytes using Cellomics high-content imaging platform. *n* = 6.

The hiPSC-derived neuronal cultures used in this study consist of a mixture of MAP2-positive neurons and GFAP-positive-astrocytes (Figure 2A). To study if both cell types had taken up the ASO equally efficiently, we determined the percentage of FAM-positive cells in both cell populations using the colocalization toolbox. As expected, of the immuno-positive cells, around 40% were neurons and 60% astrocytes. The distribution values were consistent in all conditions used and did not significantly differ from each other (Figure 2B). Increasing concentrations of the ASO using the CEM method resulted in a significant increase in the transfection efficiency of over 90% for the 500 nM concentration compared to the other conditions (Figure 2C,D). A dose-dependent increase was also seen in astrocytes. However, when comparing the transfection efficiency between neurons and astrocytes, delivery of 500 nM ASO in neurons is more effective (~90%) than in astrocytes (~55%) (Figure 2E,F).

### 3.3. Dose-Dependent CEM-Mediated ASO Uptake in D15 Brain Organoids

**Figure 3:**
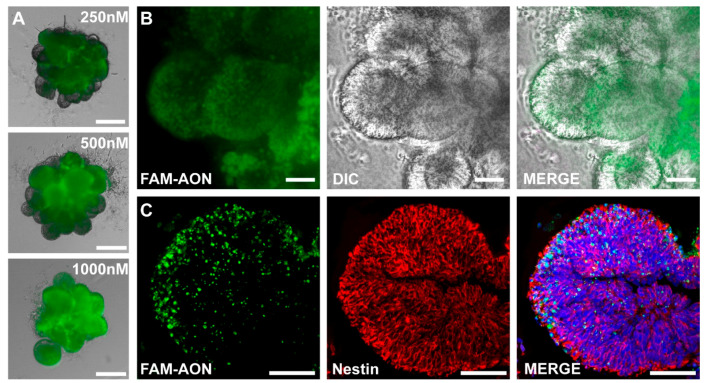
CEM-mediated ASO uptake increases in 15-day 3D brain organoids. (**A**) Representative images of D15 iPSC-derived cortical organoids treated with increasing concentrations of FAM-ASO. Scale bar 250 µm. (**B**) Representative maximum intensity projection image of D15 iPSC-derived cortical organoid treated 500 nM FAM-ASO (green), with DIC image showing structure of neural rosettes in organoid. The scale bar represents 50 µm. (**C**) Representative maximum-intensity projection image of iPSC-derived cortical organoid treated with 500 nM FAM-ASO (green) in nestin-positive (red) neural rosette structure. Scale bar 10 µm.

Human iPSC-derived 3D organoids have significantly advanced our ability to model neurodevelopmental aspects of the human brain and can provide an innovative platform for drug discovery in neurological diseases. To study the effect of novel drugs, efficient delivery is essential. Previously, it has been shown that gymnotic ASO uptake without the use of transfection agents in 3D organoids is efficient using a high concentration of ASO (10 µM) [13]. To test whether CEM-mediated ASO uptake is efficient in early-stage cortical organoids, we treated them with increasing concentrations of a FAM-labeled ASO, with the highest concentration being 1000 nM. A dose-dependent effect is seen upon increasing the concentration of the FAM-labeled ASO (Figure 3A). Representative maximum intensity projection images of D15 hiPSC-derived cortical organoids treated with 500 nM FAM-labeled ASO showed penetration of the ASO in neural rosette structures in the cortical organoid (Figure 3B). This finding was confirmed by confocal imaging of fixed sections from a representative cortical organoid treated with 500 nM FAM-ASO. The majority of the cells in Nestin-positive neural rosette structures were positive for the FAM-labeled ASO (Figure 3C).

### 3.4. Long-Term On-Target Effect of CEM-Mediated ASO Uptake in D35 Brain Organoids

**Figure 4:**
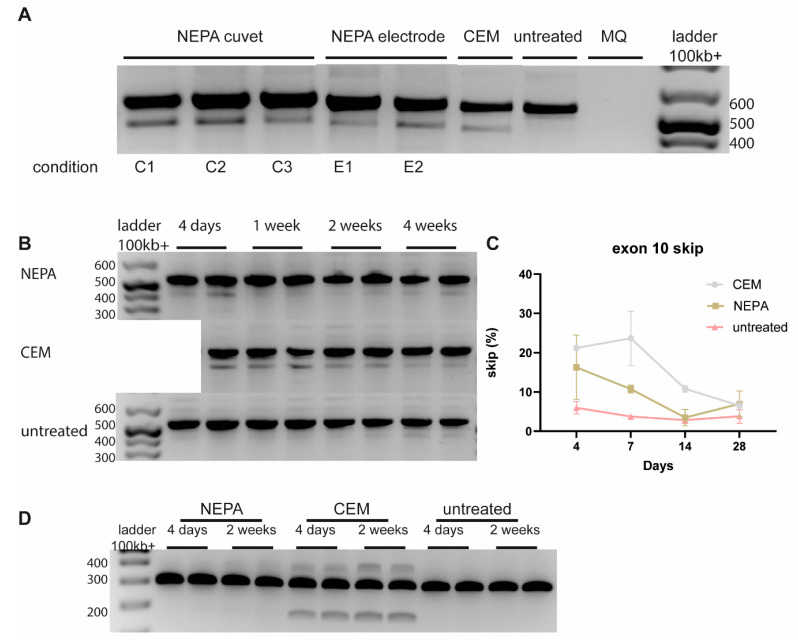
Long-term effect in 35-day 3D brain organoids. (**A**) Analysis of D35 cortical organoids showing splicing using different delivery methods using ASO12.1. (**B**) Long-term splice effect after ASO12.1 delivery using both NEPA electrode electroporation and CEM delivery. (**C**) Quantification of B. (**D**) Analysis of D35 cortical organoids showing splicing using different CEM delivery methods using an ASO targeting the APP gene. No splicing was observed by using NEPA electroporation.

Since *HTT* is only expressed high enough in D35 brain organoids, we evaluated the effect of different delivery methods of HTT-AON12.1 in D35 cortical brain organoids. Electroporation of HTT-AON12.1 using the NEPA21 electroporator showed exon skipping when either a cuvette or the NEPA electrode was used. Exon-skipping levels that were obtained were comparable to CEM delivery (Figure 4A). However, delivery with the NEPA cuvette resulted in macroscopic morphological changes in organoids directly after electroporation. To study the long-term effect after NEPA electrode and CEM delivery, we isolated RNA from cortical organoids at different time points after treatment. Here, we showed that there were consistently higher exon-skip levels with CEM delivery up to 14 days after a single ASO delivery compared to NEPA electrode delivery (Figure 4B,C). To validate these results, we studied the molecular effect of delivery of a second ASO targeting the *Amyloid Beta Precursor Protein* (*APP*) gene [17]. Here, we only found a treatment effect using CEM delivery, and the effect was stable up to 2 weeks after delivery (Figure 4D), confirming our findings that CEM delivery is more efficient than electroporation and measurable up to 2 weeks after delivery.

## 4. Discussion

The ASO field is an emerging area of therapy development that targets the disease at the RNA level rather than any downstream pathway [1,18]. To translate ASO therapeutics into clinical success, it is crucial to use standardized preclinical pipelines. The ability to differentiate iPSCs into almost all neural cell types provides a helpful tool to test ASO intervention strategies that target genes that are only highly expressed in specific neuronal cell types. One of the main hurdles to overcome in these neuronal models is ASO delivery. In our study, we found that CEM resulted in both high transfection efficiency and low toxicity in hiPSC-derived neuronal cultures. Moreover, we showed that CEM is more efficient in neurons compared to astrocytes. In brain organoids, CEM plus increasing ASO concentrations resulted in an increased ASO efficiency. Lastly, CEM has a longer effect than gymnotic uptake and electroporation.

Current in vitro delivery methods require high concentrations of ASO for gymnotic uptake or transfection reagents. Although one particular type of ASO, locked nucleic acid (LNA) phosphorothioate gapers, are easily taken up in adherent cells without the use of transfection reagents or additives, the efficiency of free-uptake varies depending on the cell line, the ASO mode of action and requires long-term exposure to medium [19]. Moreover, gymnotic uptake requires high concentrations of the ASO, which is not only expensive for screening purposes but also not in the range of a biological and physiological relevant concentration. For conventional transfection methods, one of the drawbacks is that they often involve cytotoxicity, as shown in Figure 1A. Furthermore, efficient endosomal escape is crucial for successful transfection and subsequent effect of ASOs. However, it is important to note that endosomal escape efficiency can vary depending on cell type, formulation, concentration, and specific lipofectamine reagent used. Optimization of transfection conditions, such as lipid-to-DNA ratio, incubation time, and serum concentration, can improve endosomal escape efficiency. Ca^2+^ enrichment of medium (CEM) potentiates the in vitro activity of multiple types of oligonucleotides, independent of their net charge and modifications, in various cells, including Huh-7, HLE, HeLa, HEK293, and A549 cells [12]. Microscopic analysis of cellular uptake revealed that CEM delivery provides a subcellular localization pattern of oligonucleotides similar to that achieved through unassisted uptake but with quantitative improvements [12]. When using CEM, highly monodispersed nanoparticles with a size of approximately 100 nm are observed, irrespective of the presence or absence of oligonucleotides [12]. As indicated in our study, this method has high transfection efficiency, with less cytotoxicity in hiPSC-derived neuronal cultures. Here, we show that AON delivery using CEM is more efficient in neurons compared to astrocytes in 2D hiPSC-derived neuronal cultures. So far, most of the ASO studies in animals performed a bulk analysis in specific brain regions, but recently, a single nucleus transcriptomics study on tissue from mice treated with ASOs against Prnp and Malat1 and macaques treated with an ASO against PRNP showed that activity was observed in every cell type with substantial differences in magnitude in different cell types [20]. Duration of action up to 12 weeks post-dose differed across cell types, being shorter in microglia than in neurons. Suppression in neurons was generally similar to, or more robust than, the bulk tissue [20].

Organoids offer a promising model for studying the effects of ASOs. Here, we showed a long-term on-target effect of CEM-mediated ASO uptake in brain organoids. By delivering ASOs into organoids, it becomes possible to investigate the therapeutic potential, assess target engagement, and study the cellular responses in a context that closely resembles human tissues, thereby enhancing our understanding of ASO mechanisms and optimizing the therapeutic applications. Recently, an efficient ASO transfection method to systematically evaluate and screen individual splicing events as therapeutic targets in pancreatic ductal adenocarcinoma (PDAC) organoids has been developed [21]. To improve the transfection efficiency, organoids were dissociated into single-cell suspensions and resuspended in a lipofectamine–ASO mixture. After the cells were spun down and resuspended in Matrigel using standard organoid culture conditions, high efficiency was demonstrated without destroying the characteristic organoid features [21]. A major drawback of this method is that you can only screen ASOs in early organoids that do not exhibit many of the characteristics seen in later organoids. To functionally test CEM delivery, we treated D15 cortical organoids with an increasing dose of HTT-AON12.1. A dose-dependent effect is seen upon increasing the concentration of the FAM-labeled ASO. Unfortunately, we were not able to measure the efficiency because D15 organoids only express very low levels of HTT. This stresses the importance of exploring the possibility of treating older organoids. Tynianskaia and colleagues described a fast and cost-efficient approach to genetically modify cell populations within the ventricle-like structures of primate cerebral organoids, a subtype of brain organoids. This method combines a modified protocol for the reliable generation of cerebral organoids from human-, chimpanzee-, rhesus macaque-, and common marmoset-derived iPSCs with a microinjection and electroporation approach [22]. This provides an effective tool for the study of neurodevelopmental and evolutionary processes that can also be applied to ASO therapeutics and disease modeling.

Altogether, this study shows that CEM delivery can be used as a cost-efficient and easy-to-access method for preclinical ASO screening in 2D and 3D neuronal cultures.

## Figures and Tables

**Table 1:** ASOs used in the 2D and 3D delivery experiments.

	ASO	Chemistry	Sequence
1	H40 (FAM-labeled)	2′-O-methoxyethyl-modified phosphorothioate	5′-UCC UUU CAU CUC UGG GCU C-3′
2	HTT-AON12.1	2′-O-methoxyethyl-modified phosphorothioate	5′-GUC CCA UCA UUC AGG UCC AU-3′
3	APP-AT040	2′-O-methoxyethyl-modified phosphorothioate	5′-CUU CUG CAA AGA ACA CCU UG-3′

**Table 2:** NEPA electroporation conditions.

Set Parameters											
Poring Pulse						Transfer Pulse					
V	Length (ms)	Interval (ms)	No.	D. Rate (%)	Polarity	V	Length (ms)	Interval (ms)	No.	D. Rate (%)	Polarity
225	1.5	50	4	10	+	20	50	50	5	40	+/−

**Table 3:** Primers used for RT-PCR and RT-qPCR.

	Target	Forward/Reverse primer (5′-3′)
*RT-PCR*	HTT APP	CATCACACACAGCACCAAGA/ TCTCACATCTCTGTCTGGAACC CAAGACGGAGGAGATCTCTGA/ TTGGATTTTCGTAGCCGTTC

**Table 4:** Overview of used primary and secondary antibodies.

	Manufacturer	Catalogue Number	Dilution
**Primary antibodies**			
Mouse-a-MAP2	Sigma-Aldrich, Burlington, MA, USA	MAB364	1:500
Rabbit-a-GFAP	Sigma-Aldrich Burlington, MA, USA	Z0334	1:500
Mouse-a-Nestin	Stemcell Technologies Vancouver, BC, Canada	60091	1:250
Rabbit-a-PAX6	Biolegend, San Diego, CA, USA	901301	1:250
**Secondary antibodies**			
DAPI	Sigma Aldrich, Burlington, MA, USA	D9542	1:1000
Goat-a-Mouse-Alexa594	Invitrogen, Waltham, MA, USA	A11005	1:500
Goat-a-Rabbit-Alexa594	Invitrogen, Waltham, MA, USA	A11012	1:500
Goat-a-Mouse-Alexa488	Invitrogen, Waltham, MA, USA	A11001	1:500
Goat-a-Rabbit-Alexa750	Abcam, Cambridge, UK	ab175732	1:500

**Table 5:** Overview of channels and exposure settings for the colocalization protocol in the transfection efficiency ASO experiment.

Channel	Label	Color	Wavelength	Target%	Exposure Type	Exposure Time (s)
Ch1	DAPI	Blue	386	25	Fixed	0.00612
Ch2	ASO	Green	485	25	Fixed	0.03745
Ch3	MAP2	Red	560	25	Fixed	0.02687
Ch4	GFAP	Yellow	740	25	Fixed	0.05654

## Data Availability

Data are contained within the article.
